# Supplementary material for: The interactome of intact mitochondria by cross-linking mass spectrometry provides evidence for coexisting respiratory supercomplexes
Source: Mol Cell Proteomics. 2017 Dec 8;17(2):216–32. doi: 10.1074/mcp.RA117.000470 (PMC5795388; doi:10.1074/mcp.RA117.000470)
Supplement: Supplemental Data [file supp_RA117.000470_133922_0_supp_23978_fzffwf.zip › spectra_annotation/mito_spectra_annotation/47-1-2-1-10-1.pdf]

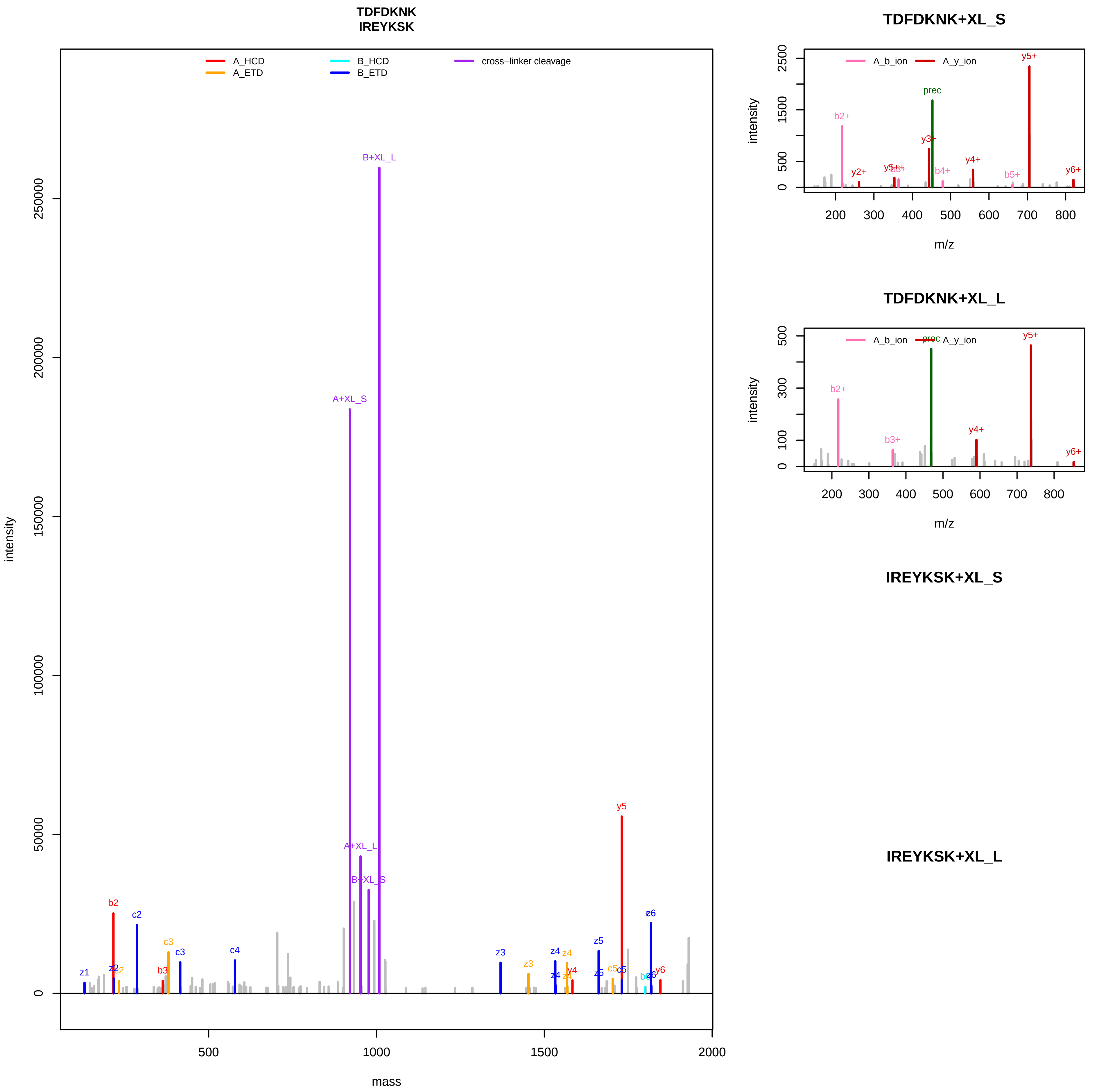

TDFDKNK
IREYKSK
A_HCD
A_ETD
B_HCD
B_ETD
cross−linker cleavage
intensity
mass
0
50000
100000
150000
200000
250000
500
1000
1500
2000
B+XL_L
A+XL_S
A+XL_L
B+XL_S
b2
c2
z1
z2
c3
b3
c4
z3
z4
z5
y5
c5
z6
y6
y4
TDFDKNK+XL_S
A_b_ion
A_y_ion
prec
b2+
y2+
y3+
y4+
y5+
b4+
b5+
y6+
m/z
TDFDKNK+XL_L
b3+
IREYKSK+XL_S
IREYKSK+XL_L
